# Type I Arnold-Chiari malformation with bronchiectasis, respiratory failure, and sleep disordered breathing: a case report

**DOI:** 10.1186/2049-6958-8-15

**Published:** 2013-02-22

**Authors:** Raffaele Campisi, Nicola Ciancio, Laura Bivona, Annalisa Di Maria, Giuseppe Di Maria

**Affiliations:** 1 Pneumology Unit Policlinico “G. Rodolico”, University of Catania, Via Santa Sofia 78, 95123, Catania, Italy; 2 Respiratory Diseases, Università di Catania, Catania, Italy; 3 School of specialization in Respiratory Dideases, University of Catania, Catania, Italy

**Keywords:** Arnold-Chiari malformation, Bronchiectasis, Central sleep apnea, Oxygen therapy, Respiratory failure, Sleep disordered breathing

## Abstract

Arnold Chiari Malformation (ACM) is defined as a condition where part of the cerebellar tissue herniates into the cervical canal toward the medulla and spinal cord resulting in a number of clinical manifestations. Type I ACM consists of variable displacement of the medulla throughout the *formamen magnum* into the cervical canal, with prominent cerebellar herniation.

Type I ACM is characterized by symptoms related to the compression of craniovertebral junction, including ataxia, dysphagia, nistagmus, headache, dizziness, and sleep disordered breathing. We report a case of a life-long non-smoker, 54 years old woman who presented these symptoms associated with bronchiectasis secondary to recurrent inhalation pneumonia, hypercapnic respiratory failure, and central sleep apnea (CSA).

CSA was first unsuccessfully treated with nocturnal c-PAP. The subsequent treatment with low flow oxygen led to breathing pattern stabilization with resolution of CSA and related clinical symptoms during sleep. We suggest that in patients with type I ACM the presence of pulmonary manifestations aggravating other respiratory disturbances including sleep disordered breathing (SDB) should be actively investigated. The early diagnosis is desirable in order to avoid serious and/or poorly reversible damages.

## Background

Arnold-Chiari malformation (ACM) is a complex syndrome in which the brainstem medulla, and the cerebellar tonsils and vermis herniate throughout the foramen magnum [1]. Type I ACM is defined by the herniation of only the medulla and cerebellar tonsils whereas type II ACM is also characterized by caudal displacement of the cerebellar vermis [2]. The main symptoms include ataxia, dizziness, chronic headache, nystagmus, twitching, oropharyngeal dysfunction, recurrent respiratory infections, paresthesia, pyramidal signs and sleep disordered breathing (SDB) encompassing a number of sleep disturbances characterized by apneas or hypopneas, intermittent hypoxaemia, microarousals, and disruption of sleep continuity [3]. All these disturbances are related to the compression of respiratory centers and their neural pathways related to herniation [4]. We report a case of type I ACM with recurrent aspiration-induced pneumonia, secondary bronchiectasis, respiratory failure, and central sleep apneas.

## Case report

**Figure 1:**
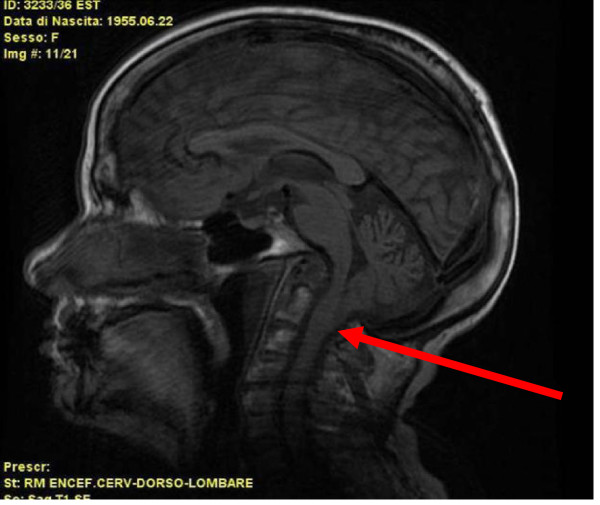
**Sagittal craniocervical magnetic resonance imaging section. **The arrow shows cerebellar tonsils herniation trough the foramen magnum until the second cervical vertebra and bulbar compression.

**Figure 2:**
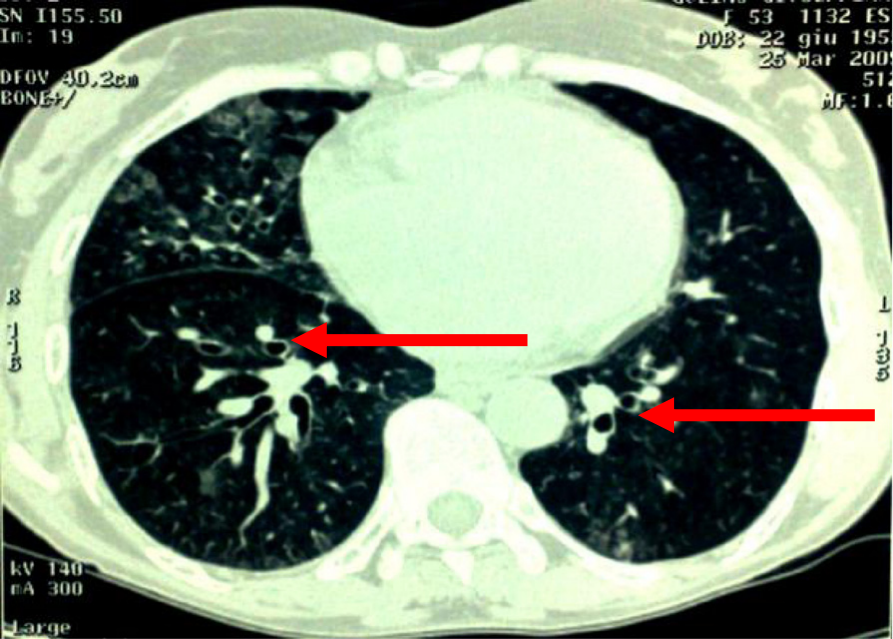
**Bronchiectasis. **The arrows show cylindrical bronchiectasis spread to both lungs appearing as “signet ring”.

**Figure 3:**
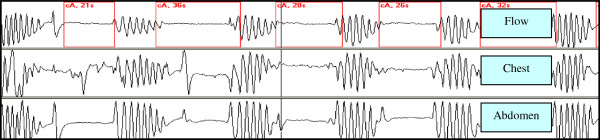
**Central sleep apnoeas. **Flow, chest and abdomen pattern during central sleep apnoeas.

A 54-years-old woman with type I ACM (BMI 19.2 kg/m^2^, neck and waist circumference 34 and 65 cm respectively, Mallampati score 2), was referred to our Respiratory Unit with a history of chronic cough and purulent sputum, fever, intense dyspnoea (MRC dyspnoea scale 4), hoarseness, excessive daytime sleepiness (Epworth Sleepiness Scale 14), involuntary naps, snoring, nocturia and morning headaches. These respiratory symptoms had been present for more than five years. She referred six hospital admissions because of inhalation pneumonia in the last five years. Physical examination revealed normal pulse rate (66 per minute), high respiratory rate (24 per minute), normal blood pressure (115/70 mmHg) and low oxygen saturation (SpO_2_ 90%). Cardiac auscultation was normal, whereas pulmonary auscultation revealed diffuse rales in both lungs, basal and bilateral crackles. Neurologic examination showed nistagmus, tongue twitching, dysarthria, dizziness, walking ataxia, severe dysphagia and persistent bilateral abductor vocal cord paralysis. Routine blood tests gave normal results apart from high level of C-reactive protein (5.68 mg/dl), ESR (64 mm) and low serum albumin (3.3 g/dl). Cervico-medullary magnetic resonance imaging showed cerebellar tonsils herniation through the foramen magnum to the level of C2 vertebra, basilar imprint and bulbar compression [Figure 1]. Chest X-Ray showed no pulmonary consolidation whereas several cylindrical bronchiectasis of both lungs were seen on chest CT [Figure 2]. All causes for congenital bronchiectasis were excluded. The patient had no family history of cystic fibrosis, α1-antitrypsin deficiency, or other conditions that predispose to the onset of secondary bronchiectasis such as tuberculosis or infection during childhood. Sputum smear for acid fast bacilli and Quantiferon-TB test were negative. Arterial PO_2,_ PCO_2_ and pH were 58, 50 mmHg, and 7.43 respectively. Pulmonary function test revealed volumes within normal limits [FVC 2,13 L (88% pred), FEV_1_1,67 L (93% pred), FEV_1_/FVC ratio 0.78. The diffusion capacity of the lung for CO (DLco): 103% pred; DLco/V_A_ 1.47 (110% pred.). Because of excessive daytime sleepiness, heavy snoring and hypercapnic respiratory failure we performed overnight polysomnography in room air, after titration of c-PAP (9 cm H_2_O), and during nasal oxygen administration (FiO_2_ 0,24%) overnight. These consisted of recording of airflow, pulse oximetry, thoracic and abdominal movements, heart rate, body position, snoring, legs positions and two-channel electroencephalogram. Apneas, hypopneas, and apnea-hypopnea index (AHI) were defined according to current criteria [5,6]. Polysomnography in room air revealed an AHI of 42 events h^-1^ with several prolonged episodes of Central Sleep Apneas (CSA) and some events of obstructive sleep apnea (OSA) and an average of arterial saturation of 89% [Figure 3]. The patient did not show any cardiovascular comorbidity (normal echocardiogram, no signs of pulmonary hypertension or cardiac arrhythmias).

After titration of c-PAP , polysomnography showed a decrease in AHI from 42 to 22 events h^-1^ and mean of SpO_2_ was 92%. Registration during nasal oxygen administration revealed a reduction in the number of both CSA and OSA allowing a significant improvement of AHI along with apnoea duration; mean SpO_2_ was 96% [Table 1]. Capnography was performed to evaluate changes in nocturnal hypercapnia. Average pCO_2_ was 52 mmHg (range 44–56 mmHg). Average SpO_2_ was 89%. During low-flow nocturnal oxygen (FiO_2_ 0,24%) pCO_2_ was 57 mmHg (range 53-63 mmHg); average SpO_2_ was 96%. During wakefulness the patient returned to be hypercapnic with values similar to baseline, then worsening of average pCO_2_ was only partial and transitory. The patient did not take any drug known to alter the sleep pattern. Administration of broad-spectrum antibiotics (piperacillin/tazobactam 4,5 g/t.i.d and Ciprofloxacin 500 mg b.i.d.] was started, and corticosteroid (prednisone 25 mg/die) was administered for 7 days. During follow up examination, the patient was clinically improved, with regression of cough, sputum, improvement in respiratory symptoms and daytime sleepiness (ESS 5), but persistent hypercapnic chronic respiratory failure.

**Table 1:** Polysomnographic data at baseline, during C-PAP breathing, and during continuous oxygen administration

**Polysomnographical data**			
	**Baseline**	**C-PAP**	**FiO2 24%**
**Polysomnography**			
Total Sleep Time	7 h 03 m	7 h 40 m	7 h 30 m
Sleep onset latency	18.8 m	21.3 m	18.8 m
% Sleep efficiency	89	90	92
% Slow Wave Sleep	27	31	33
% REM	19	22	25
**Respiratory Events**			
AHI	42	22	1
AC	125	63	3
AO	1	9	1
AM	9	0	0
HI	186	77	1
CH	175	72	3
OH	11	5	0
O2 saturation	89%	92%	96%
ODI	43	15.7	3.0
cT90	30%	21%	0%
Nadir SaO2	82	84	90

*AHI* Apnoea/hypopnoea index, *AC* Central apnea, *AO* Obstructive apnoea, *AM* Mixed apnoea, *HI* Hypopnoea; *CH* Central Hypopnoea, *OH* Obstructive Hypopnoea, *ODI* Oxygen Desaturation Index, *cT90* percentage of sleep time spent with SaO2 below the threshold of 90%; SaO2: arterial saturation; *REM* Rapid Eye Movements.

## Discussion

This case presentation offers the opportunity to speculate about the occurrence of respiratory involvement and its mechanisms in patients with type I ACM. Sleep disordered breathing is associated with ACM and generally ascribed to two types of abnormalities: upper airway dysfunctionwhich is associated with obstructive apneas, and abnormalities of respiratory controlwhich is presumably involved in the pathophisiology of central sleep apneas [4,7]. The latter are characterized by transient cessation of neural respiratory output during sleep resulting in poor ventilation and impaired gas exchange [8]. The transient cessation of respiratory drive could be due to: firstly, an outright defect in respiratory drive; secondly, a transient instability in an otherwise intact respiratory control system; and thirdly, a transient active inhibition of respiratory motor drive [9]. In addition, patients may be either hypercapnic or non-hypercapnic. The hypercapnic group, which includes patients with central hypoventilation and a number of neurological syndromes, is consistent with the first pathophysiological mechanism. The non-hypercapnic group includes patients with idiopathic hyperventilation and periodic breathing. Pathophysiologically this group is consistent with the second or third mechanism. These patients typically have a low or normal awake pCO_2_[10]. In OSA, pharyngeal anatomy, upper airway muscles responsiveness during sleep, arousal threshold, and loop gain may all contribute to the occurrence of apnea presence and severity of central apneas. During sleep reflex muscles activation is reduced and if the airway anatomy is quite deficient will likely lead to substantial or complete airflow obstruction, yielding a hypopnoea or apnea [11]. The patient had the hypercanic form of CSA The risk of apnoea resulted from both obstructive (short neck, limited mobility of soft palate and tongue) and central causes. Central causes may include: 1) compromised vascular supply to the brainstem due to compression; 2) insensitivity of peripheral chemo-receptors, due to brainstem involvement; 3) direct compression of the respiratory centre [11]. In our case both central and obstructive apneas was confirmed with polysomnography. Nasal c-PAP (9 cmH2O), after proper titration, was partially effective in improving the AHI and apnea duration, but limited compliance to the treatment. Polysomnography was repeated during low flow oxygen administration resulting in a significant reduction in both number and duration of CSA and an increase in SpO_2_ (average apnea duration in baseline condition 16.5 seconds; after low flow oxygen administration 11.2 sec). The use of supplemental low flow oxygen, as mentioned in another case report of a patient with primary alveolar hypoventilation, chronic hypercapnia and CSA, led to a decrease in number and duration of central apneas [11,12]. The improvement produced by oxygen may have been due to the fact that the patient had no demonstrable ventilatory response to hypoxia during wakefulness, and therefore may have developed hypoxic brainstem depression during sleep. The findings suggest that oxygen therapy during sleep may be beneficial in patients with primary alveolar hypoventilation and CSA leading to significant improvement of SDB and all related symptoms [12]. Oxygen administration during sleep has been associated with reproducible reduction of AHI [Table 1] Type I ACM**,** whether alone or in combination with syringomyelia, can cause a great number of progressive disorders such as dysphagia, alveolar hypoventilation, inhalation pneumonia, and respiratory failure [13]. In our patient recurrent aspirations with consequent inhalation pneumonia occurred. The most important mechanism of recurrent aspiration pneumonia was dysphagia [14]. The alterations underlying dysphagia are stretch injury to the lower cranial nerves caused by caudal displacement of the medulla or compression of the swallowing centres in the brainstem [15]. Probably the pressure determined by the cerebellar tonsils on the hypoglossal nuclei and other swallowing centres located in the medulla is tough to be the leading cause of the dysphagia [16]. Recurrent aspirations result in several respiratory infections which may lead both to post-inflammatory bronchiectasis and lung parenchymal damage, causing chronic respiratory failure (CRF) [17,18]. Respiratory failure as the early manifestation in type I ACM is uncommon and, generally, is the result of postoperative conditions [19]. Cylindrical bronchiectasis, as documented in Figure 2, have become a source of repeated infections with recurrent exacerbations of CRF, chronic cough, intense dyspnoea and fever treated with antibiotics and often requiring hospitalization [20]. Respiratory failure probably has been caused not only by neuromuscular disorders affecting the diaphragm due to compression of neural centers in the brainstem, but also resulted from swallowing disturbances and dysphagia further complicated by recurrent aspiration pneumonia.

## Conclusions

This case report suggests that a neurologic cause can always be considered for recurrent aspiration pneumonia and progressive dysphagia, even in absence of prominent signs and symptoms. The high prevalence of sleep apnoea syndrome in patients with neurological disorders indicates that respiratory disturbances during sleep should be systematically screened even in ACM patients, in order to prevent nocturnal respiratory failure and all the risks associated with nocturnal intermittent hypoxia. In summary, central sleep apnoea can be the typical manifestation of ACM and may be a life-threatening condition. The severity of CSA may explain the reported increased incidence of death during sleep in ACM patients [11]. Using a low flow oxygen during sleep, even in hypercanic patients, avoids mechanical ventilation that is often not well tolerated. Oxygen administration allows to solve CSA and all related cerebrovascular risks associated with nocturnal respiratory failure and sleep fragmentation, improving quality of life. Oxygen therapy can generate potentially deprimental effects. The most relevant of these is the worsening of hypercapnia, which is mediated by mechanisms such as hypoventilation and ventilation-perfusion redistribution. Particularly sleep itself generates ventilatory alterations that include an increase in airway resistance and decreased sensitivity of respiratory centers. Arterial blood gases samples should be periodically taken at awakening to assess pCO_2_ in order to prevent hypoventilation from the oxygen therapy [21,22]. The availability of further clinical studies for the treatment of CSA with low flow oxygen in hypercapnic patients is desiderable to avoid serious and irreversible damage.

## Consent

"Written informed consent was obtained from the patient for publication of this Case report and any accompanying images. A copy of the written consent is available for review by the Editor-in-Chief of this journal."

## Abbreviations

ACM: Arnold Chiari Malformation;AHI: Apnea/Hypopnea Index;CRF: Chronic Respiratory Failure;CSA: Central Sleep Apnea;OSA: Obstructive Sleep Apnea;SDB: Sleep Disordered Breathing

## Competing interests

All the author declare that they have no competing interest.

## Authors’ contributions

RC conceived and drafted the manuscript and the study design. NC, LB and ADM participated in the design of the study. GDM participated in the design and coordination of the study and helped to draft the manuscript. All authors read and approved the final manuscript.
